# Triplets versus doublets, with or without cisplatin, in the first-line treatment of stage IIIB–IV non-small cell lung cancer (NSCLC) patients: a multicenter randomised factorial trial (FAST)

**DOI:** 10.1038/bjc.2011.606

**Published:** 2012-01-12

**Authors:** C Boni, M Tiseo, L Boni, E Baldini, F Recchia, C Barone, F Grossi, D Germano, E Matano, G Marini, R Labianca, F Di Costanzo, A Bagnulo, C Pennucci, C Caroti, M Mencoboni, F Zanelli, T Prochilo, M A Cafferata, A Ardizzoni

**Affiliations:** 1 Oncology Unit, S Maria Nuova Hospital, Viale Risorgimento 80, Reggio Emilia 42123, Italy; 2 Oncology Unit, University Hospital, Via Gramsci 14, Parma 43100, Italy; 3 Clinical Trials Coordinating Center, Istituto Toscano Tumori, University Hospital Careggi, Largo Brambilla 3, Firenze 50134, Italy; 4 Oncology Unit, S Chiara University Hospital, Via Roma 67, Pisa 56100, Italy; 5 Oncology Unit, Civil Hospital, Via G Di Vittorio 36, Avezzano 67051, Italy; 6 Oncology Unit, A Gemelli University Hospital, Largo Gemelli 8, Roma 00168, Italy; 7 Oncology Unit A, National Institute for Cancer Research, Largo R Benzi12, Genova 16132, Italy; 8 Oncology Unit, S Carlo Hospital, Via Potito Petrone, Potenza 85100, Italy; 9 Oncology Unit, Federico II University Hospital, Via Sergio Pansini 5, Napoli 80131, Italy; 10 Oncology Unit, Azienda Ospedaliera Spedali Civili di Brescia, Via Camillo Biseo 17, Brescia 25128, Italy; 11 Oncology Unit, Riuniti Hospital, Largo Giovanni Barozzi, Bergamo 24128, Italy; 12 Oncology Unit, University Hospital Careggi, Largo Brambilla 3, Firenze 50134, Italy; 13 Oncology Unit, San Sebastiano Hospital, Via Mandriolo Superiore 11, Correggio 42015, Italy; 14 Oncology Unit, Civil Hospital, Piazza Sacco e Vanzetti, Carrara 54033, Italy; 15 Oncology Unit, Galliera Hospital, Mura delle Cappuccine 14, Genova 16128, Italy; 16 Oncology Unit, Villa Scassi Hospital, Corso Onofrio Scassi 1, Genova 16149, Italy

**Keywords:** doublet, chemotherapy, cisplatin, non-small cell lung cancer (NSCLC), triplet

## Abstract

**Background:**

The FAST is a 2 × 2 factorial trial addressing two questions: (1) the role of replacing cisplatin (P) with a non-platinum agent, vinorelbine (N), and (2) the role of adding a third agent, ifosfamide (I), in a doublet based on gemcitabine (G).

**Methods:**

A total of 433 stage IIIB–IV non-small cell lung cancer (NSCLC) patients were randomised to one of four arms: gemcitabine–cisplatin (GP), gemcitabine–vinorelbine, gemcitabine–ifosfamide-cisplatin or gemcitabine–ifosfamide–vinorelbine. Two comparisons were performed: N- *vs* P-containing regimens and I-triplets *vs* non-I doublets.

**Results:**

For N- *vs* P-containing regimens, adjusted overall survival was 9.7 *vs* 11.3 months (*P*=0.044), progression-free survival was 4.9 *vs* 6.4 months (*P*=0.020) and response rate was 24% *vs* 31% (*P*=0.124), respectively. No statistically significant difference was observed between doublets and triplets. Grade 3–4 haematological toxicity was significantly more frequent in P-containing therapy; grade 3–4 leucopenia was significantly more common in triplets. Concerning non-haematological toxicity, grade 3–4 nausea-vomiting was significantly increased in P-containing regimens.

**Conclusions:**

This trial provides evidence of a slight survival superiority of GP-containing regimens over platinum-free N-containing chemotherapy. This trial also confirms that the addition of a third chemotherapy agent (I) to a standard G-based doublet does not improve treatment outcome.

Until recently, the standard treatment of advanced non-small cell lung cancer (NSCLC) has been based on a combination of cisplatin with a third-generation chemotherapic agent, such as paclitaxel, docetaxel, vinorelbine or gemcitabine (Azzoli *et al*, 2009; D’Addario *et al*, 2010). These regimens obtained comparable outcome results (Kelly *et al*, 2001; Scagliotti *et al*, 2002; Schiller *et al*, 2002) but, among these, at least in Europe, cisplatin–gemcitabine has been the most used combination (Le Chevalier *et al*, 2005).

At the time this trial was designed the discussion concerning optimal chemotherapy treatment of advanced NSCLC was mainly focused on the uncertain superiority of platinum- *vs* non-platinum-based regimens and of triplets *vs* doublets.

Despite its pivotal role in NSCLC management, cisplatin is associated with a number of serious side effects including nausea-vomiting, neurotoxicity and renal function impairment and it is burdened by delivery problems such as the need for prolonged hydration (Tiseo *et al*, 2007). To overcome these limitations, most clinicians were considering the use of carboplatin and of platinum-free combinations as a possible alternative.

Although carboplatin and cisplatin have similar mechanism of action and spectrum of activity, some trials and an individual patient data meta-analysis evidenced that cisplatin-based is slightly superior to carboplatin-based chemotherapy in terms of response rate (RR) and also, in certain subgroups (third-generation regimens and non-squamous histology), in terms of survival, without a significant increase in severe toxicity (Ardizzoni *et al*, 2007).

The activity and tolerability of third-generation agents led many investigators to evaluate platinum-free doublets in the hope that platinum analogues could be spared for the treatment of advanced NSCLC (Georgoulias *et al*, 2001; Kosmidis *et al*, 2002; Gridelli *et al*, 2003).

Addition of a third agent to platinum-based doublet may be an option to improve outcomes in NSCLC. This strategy has been shown to be associated with superior outcomes in other malignancies (Vermorken *et al*, 2007). This led to the conduct of multiple trials comparing a two-drug regimen with a three-drug regimen in advanced NSCLC patients (Comella *et al*, 2000; Alberola *et al*, 2003; Laack *et al*, 2004; Paccagnella *et al*, 2006). Our group, in particular, developed two different triplets including ifosfamide, an alkylating agent with activity against NSCLC commonly used in old regimens. Gemcitabine, ifosfamide and cisplatin (GIP) and gemcitabine, ifosfamide and vinorelbine (GIN) evidenced very interesting results in phase II first-line studies, with a RRs of 54% and 52% and a median overall survival (OS) of 12 and 11 months, respectively, with acceptable toxicity profiles (Boni *et al*, 2000; Baldini *et al*, 2001). The results of these studies suggested further investigations within prospective randomised study assessing the role of these triplets, with or without platinum.

Considering this background, a randomised 2 × 2 factorial phase III trial addressing two questions: (1) the role of replacing cisplatin (P) with a non-platinum agent, vinorelbine (N), and (2) the role of adding a third agent, ifosfamide (I), in a chemotherapy doublet based on gemcitabine (G) was performed. Here, the results of this multicenter Italian trial are reported.

## Patients and methods

### Study population

Patients with histologically or cytologically confirmed locally advanced stage IIIB (supra-clavicular node and/or malignant plural effusion) or metastatic stage IV (according sixth TNM classification) NSCLC were eligible for the study. Patients were required to be chemotherapy-naive for advanced disease. Eligibility criteria included: age ⩾18 years, ECOG performance status (PS) ⩽2, adequate haematological, hepatic and renal function. Patients with active infection, severe co-morbidity and a history of previous or concomitant neoplasm, other than epithelial tumours of the skin or *in situ* carcinoma of the uterine cervix, were ineligible.

The protocol was conducted in accordance with the Declaration of Helsinki and Good Clinical Practice guidelines. The study was approved by the ethics committee of each participating institution and written informed consent was obtained from each patient before inclusion.

### Study design and treatment plan

This was a randomised factorial study with the following two primary aims: (1) to compare the effectiveness of two different treatment strategies, one containing cisplatin and one containing vinorelbine instead of cisplatin; (2) to compare the effectiveness of two different treatment strategies, one with two and one with three drugs for the addition of ifosfamide, both in terms of OS.

The factorial design was chosen to improve study efficiency, assuming no interaction between the two factors under investigation.

After stratification by centre, eligible patients were randomly assigned to one of four treatment arms in a 1 : 1 : 1 : 1 ratio: gemcitabine–cisplatin (GP), gemcitabine–vinorelbine (GN), GIP and GIN. Random assignment was centrally performed by fax at the Trial Unit of the National Institute for Cancer Research of Genova with the use of permuted blocks of variable size.

The GP regimen consisted of: gemcitabine 1250 mg m^–2^ on days 1 and 8 and cisplatin 80 mg m^–2^ (infused with hydration) on day 1 every 21 days. The GN regimen consisted of: gemcitabine 1250 mg m^–2^ on days 1 and 8 and vinorelbine 25 mg m^–2^ on days 1 and 8 every 21 days. The GIP regimen consisted of: gemcitabine 1000 mg m^–2^ on days 1 and 8, ifosfamide 2 g m^–2^ (with mesna total dose of 1200 mg administered as an i.v. bolus immediately before ifosfamide infusion and after 4 and 8 h) on day 1 and cisplatin 80 mg m^–2^ (infused with hydration) on day 1 every 21 days. The GIN regimen consisted of: gemcitabine 1000 mg m^–2^ on days 1 and 8, ifosfamide 3 g m^–2^ (with mesna total dose of 1600 mg administered as an i.v. bolus immediately before ifosfamide infusion and after 4 and 8 h) on day 1 and vinorelbine 25 mg m^–2^ on days 1 and 8 every 21 days.

Clinical examination was performed before every cycle and 21 days from the end of therapy. Complete blood cell count was performed on day 1 and between days 12 and 14.

Serum liver and renal functions were measured before each cycle of chemotherapy and at the end of the treatment. Dose reductions of single drugs and delay of each cycle were applied according to standard criteria defined by protocol schedules. Primary prophylaxis with G-CSF was not allowed. The treatment was given for a maximum of six cycles unless there were disease progression, unacceptable toxicity or withdrawal of the consent.

### Statistical analyses

The primary end point was OS. Secondary end points included characterisation of toxicities, objective RR and progression-free survival (PFS). The study was designed to detect a 25% relative reduction of the mortality hazard, in both planned comparisons (N-based *vs* P-based regimens and three-drug *vs* two-drug regimens). We aimed to enrol enough patients to yield the occurrence of 385 deaths, which would give a statistical power of 80% to reject the null hypothesis of no significant difference in the OS time in the two planned comparisons, assuming a hazard ratio (HR) of 0.75, a significance level of a two-sided log-rank test fixed at 5%, an accrual rate equal to 230 patients per year and a minimum follow-up duration of 2 years. No adjustment for multiple comparisons was made.

Overall survival was measured from the date of randomisation to the date of death from any cause. Progression-free survival was measured from the date of randomisation to the first date of disease progression or of death from any cause. In both OS and PFS analyses, observation times were censored at the limit date of 30 September 2009 for patients in whom no event occurred.

Objective response (complete and partial response) was evaluated according to RECIST criteria (version 1.0) (Therasse *et al*, 2000). Response was assessed after three and six courses with a CT scan. The best overall response is the best response recorded from the start of treatment until disease progression. Patients who received at least one dose of chemotherapy were considered evaluable for response; any patient who died early, had early suspension of chemotherapy because of any cause or was not evaluated after randomisation was considered non-responder.

Toxicity grading, based on NCIC–CTC toxicity criteria (version 2.0, National Cancer Institute of Canada Common Toxicity Criteria, Kingston, ON, Canada), was evaluated weekly. All efficacy analyses were based on the intention-to-treat (ITT) principle. Safety was analysed on all subjects receiving at least one dose of study drugs, according to treatment actually received (safety population).

Median period of follow-up was calculated for the entire study cohort according to the reverse Kaplan–Meier method. Non-parametric estimates of the survivor functions and hazard ratios were based on the Cox proportional hazards model, using the average covariate method, and each of them was adjusted by the other treatment factor (presence of platinum and number of drugs). Confidence intervals (CIs) of median survival times were calculated according to the log–log method of Brookmeyer and Crowley (1982). Adjusted estimates of objective RRs and ORs with their 95% CIs were obtained by a logistic regression model. Wald *χ*^2^-test was used to test the statistical significance of all coefficients. All reported *P*-values are two-sided and significant level was set at ≤0.05. Efficacy analyses were performed whenever the results did not suggest the presence any interaction between the two different treatment modalities. Statistical analyses were performed by LB at Istituto Toscano Tumori using SAS System 9.2 (Cary, NC, USA) and R statistical packages.

## Results

**Figure 1:**
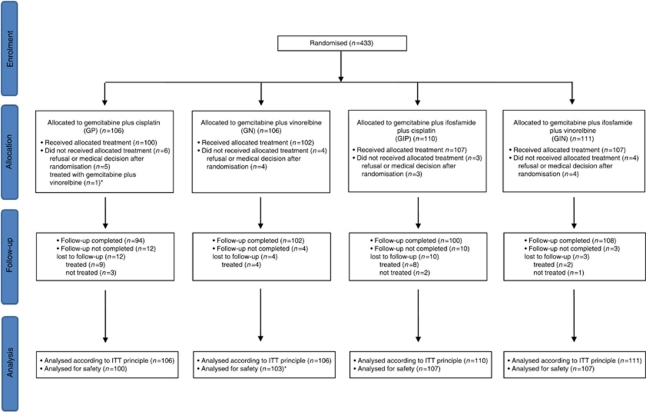
CONSORT diagram of the study. A total of 417 patients (96.3%) received study treatment consisting of at least one dose of chemotherapy. ^*^One patient was assigned to the GP arm but received GN treatment. This patient was included in the GN arm for the safety analysis. GP, gemcitabine–cisplatin; GN, gemcitabine–vinorelbine; GIP, gemcitabine–ifosfamide–cisplatin; GIN gemcitabine–ifosfamide–vinorelbine.

Figure 1 summarises patient disposition in the trial. From October 2001 to July 2006, a total of 433 stage IIIB–IV NSCLC patients were enrolled and randomly assigned to one of the four treatment regimens: GP (*n*=106), GN (*n*=106), GIP (*n*=110) and GIN (*n*=111), at 15 participating Italian centres. Therefore, 216 patients were expected to receive P-based therapy (GP and GIP) and 217 N-based regimen (GN and GIN), whereas 212 patients were expected to be treated with a doublets (GP and GN) and 221 with a triplets (GIP and GIN). Of these 433 patients, 16 (3.7%) did not receive any study therapy; thus, 417 patients are included in the safety analysis population. Six (1.4%) out of 433 patients with non-measurable disease at randomisation were excluded from the analysis of best overall response.

### Patient characteristics

As shown in Table 1, patient characteristics for ITT population at baseline were well balanced between the two groups in both comparisons. The median age of patients was 63 years (range of 29–79). Most patients in all arms were male and had an ECOG PS of 0. In all, 80% of patients in all treatment groups had stage IV or recurrent disease and 27% had squamous histology. Supplementary Table S1 reported baseline patient and tumour characteristics by allocated treatment arm.

### Therapy administration and toxicity

The mean and median number of treatment cycles administered were 4.45 and 5 (range 1–6), respectively, in patients treated with GP and GIP and 4.19 and 4 (range 1–6), respectively, for those who received GN and GIN. In the second comparison, the mean and median were 4.37 and 5 (range 1–6), respectively, for patients treated with doublets and 4.27 and 4.5 (range 1–6), respectively, for patients treated with triplets.

Grade 3 and 4 haematologic and non-haematologic toxicities exceeding 5% of the treated patients are reported in Table 2. Haematological toxicity consisted mainly in grade 3–4 anaemia (14% *vs* 5% *P*=0.001), leucopenia (33% *vs* 23% *P*=0.025) and thrombocytopenia (32% *vs* 4% *P*<0.001), significantly more frequent in P-containing *vs* N-containing regimens. Also febrile neutropenia was significantly more frequent in patients treated with P-containing *vs* N-containing regimens (3% *vs* 0.5% *P*=0.044). The triplets were more frequently responsible of grade 3–4 leucopenia (35% *vs* 22% *P*=0.003) than doublets.

Concerning non-haematological toxicity, grade 3–4 nausea-vomiting (12% *vs* 4% *P*=0.004) was significantly increased in P-containing regimens compared with N-therapy; no other statistically significant differences in toxicity were observed in both comparisons (Table 2).

Supplementary Table S2 reported grade 3 and 4 haematologic and non-haematologic toxicities exceeding 5% of the treated patients by allocated treatment arm.

### Efficacy

**Figure 2:**
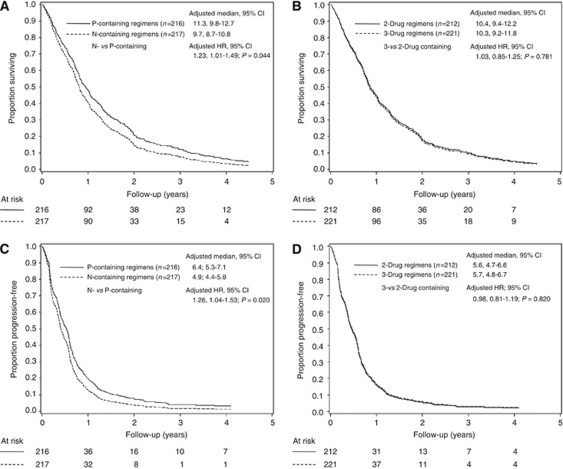
Kaplan–Meier overall survival (OS) and progression-free survival (PFS) curves for two comparisons. (**A**, **C**) N-containing versus P-containing regimens; (**B**, **D**) 3- versus 2-drug regimens. N, vinorelbine; P, cisplatin.

Primary efficacy outcomes by two comparisons are summarised in Table 3. Supplementary Table S3 reported response and survival outcomes by allocated treatment arm. Median follow-up was 66.4 months (interquartile range: 49.7–67.5). A total of 29 patients (6.7%) were lost to follow-up. Curves for OS and PFS are summarised in Figure 2 for the two comparisons.

Median OS was 10.3 months for all 433 patients. Adjusted median survival was 11.3 months (95% CI: 9.8–12.7) *vs* 9.7 months (95% CI: 8.7–10.8) favouring P-containing therapies (HR)=1.23; 95% CI: 1.01–1.49; *P*=0.044) (Figure 2A). Adjusted median survival was 10.4 months (95% CI: 9.4–12.2) and 10.3 months (95% CI: 9.2–11.8) for doublets and triplets, respectively (HR = 1.03; 95% CI: 0.85–1.25; *P*=0.781) (Figure 2B). There was no statistically significant interaction between the trial arms (*P*=0.146).

Adjusted median PFS was 6.4 months for P-containing regimens (95% CI: 5.3–7.1) and 4.9 months for N-containing therapies (95% CI: 4.4–5.8; HR=1.26; 95% CI: 1.04–1.53; *P*=0.020) (Figure 2C). Adjusted median PFS was 5.6 months for doublets (95% CI: 4.7–6.6) and 5.7 months for triplets (95% CI: 4.8–6.7; HR=0.98; 95% CI: 0.81–1.19; *P*=0.820) (Figure 2D). No evidence of multiplicative interaction between treatment arms was observed (*P*=0.073).

Adjusted overall RR (complete and partial responses) were 31% *vs* 24% for platinum *vs* non-platinum therapies, respectively (OR=0.72; 95% CI: 0.47–1.10; *P*=0.124), and 29% *vs* 26% for doublets and triplets, respectively (OR=0.86; 95% CI: 0.56–1.32; *P*=0.487) (test for interaction between trial arms: *P*=0.748).

### Additional analyses

Clinical results were retrospectively analysed by patient histology (squamous *vs* non-squamous tumours). The RR in non-squamous patients was numerically greater than that achieved in squamous (30% *vs* 23% *P*=0.151). Median survival was 10.8 months (95% CI: 9.7–12.4) and 9.4 months (95% CI: 8.4–10.4) for non-squamous and squamous patients, respectively (HR=1.25; 95% CI: 1.00–1.56; *P*=0.048). Median PFS was 5.8 months (95% CI: 4.9–6.7) and 4.9 months (95% CI: 4.3–6.0) for non-squamous and squamous patients, respectively (HR=1.18; 95% CI: 0.95–1.46; *P*=0.136).

No evidence of interaction effect between histological subtype and treatment type (P- *vs* N-containing regimens) was observed on RR, PFS and OS (*P*=0.943, 0.923 and 0.542, respectively).

## Discussion

This prospective, factorial randomised trial is the only single study in advanced NSCLC treatment demonstrating a statistically significant slight superiority in survival of platinum-containing regimens over platinum-free chemotherapy. On the contrary, this trial does not provide evidence of an improved outcome with the addition of a third chemotherapy agent, such as ifosfamide, to a standard doublet.

At the time this trial was designed, the controversy about standard chemotherapy treatment of advanced NSCLC was mainly concentrated on the role of platinum and on the optimal number of agents to be used. Therefore, we designed this study aimed at answering with a single study these two relevant questions. Considering, cisplatin–gemcitabine as one of the most widely used platinum-based doublets for the treatment of advanced NSCLC in EU, the available data about GN combination (Gridelli *et al*, 2000) and the previous experience of our group with two different triplets (GIP and GIN) (Boni *et al*, 2000; Baldini *et al*, 2001), we included these four treatment arms in a 2 × 2 factorial study design.

Results from this trial are largely consistent with the data, which have been subsequently available in the literature about the first-line treatment of NSCLC patients and, also, with International guidelines (Azzoli *et al*, 2009; D’Addario *et al*, 2010).

No previous study was successful in demonstrating a statistically significant survival advantage in favour of platinum-based regimens, compared with platinum-free combinations. However, in some studies, a trend towards a higher RR or PFS or OS was observed in patients treated with platinum-based combinations compared with those treated with cisplatinum-free regimens (Georgoulias *et al*, 2001, 2004; Kosmidis *et al*, 2002; Alberola *et al*, 2003; Gridelli *et al*, 2003; Smit *et al*, 2003; Kubota *et al*, 2008). D’Addario *et al* (2005) reported the results of a meta-analysis based on abstracted data from randomised phase II and III studies designed to compare the efficacy and toxicity of platinum-based to non-platinum-based chemotherapy in advanced NSCLC. As in our trial, the platinum-based combinations had a significantly higher 1-year survival rate compared with the non-platinum regimens.

The results of our study concerning this comparison are consistent also with the results of two other meta-analysis of phase III trials comparing third-generation platinum- *vs* non-platinum-based combinations (Pujol *et al*, 2006; Rajeswaran *et al*, 2008). Pujol *et al* (2006) showed that patients treated with platinum-based doublets had a statistically significant reduction in the risk of death (OR=0.88, *P*=0.044) without an unacceptable increase in toxicity. Moreover, Rajeswaran *et al* (2008) confirmed that cisplatin-, but not carboplatin-based regimens, are associated with a slight survival advantage at 1-year compared with non-platinum-based doublets.

In our study, the absolute benefit at 1-year turns out to be relatively small, but it becomes more relevant later, as shown in the Kaplan–Meier curve, with a higher percentage of long-term surviving patients in the P-containing arms. This result is reinforced by the high median follow-up time (66.4 months) of our study. This is in keeping with the hypothesis that the added benefit of platinum, albeit quantitatively small, may translate into a long-term higher survival probability.

P-containing regimens showed, moreover, a short-term benefit in PFS, about 2 months in median PFS and 6.8% absolute improvement in 1-year PFS probability, without any statistically significant difference in RR. This observation might be probably related to the high percentage (22%) of patients not evaluable for response and to the lack of uniform external and blinded radiological response assessment.

One of the most remarkable results of the study was the fairly good tolerability of all the four regimens, with a slightly higher haematological toxicity in P-containing regimens, which were penalised also by a higher incidence of nausea-vomiting.

Also the triplets were well tolerated, even if with significantly more grade 3–4 leucopenia compared with doublets. According to previous results, also in our trial the addition of a third agent to doublet therapy showed no survival benefit (Delbaldo *et al*, 2004). In interpreting these results, however, it has to be reminded that doses of both gemcitabine and ifosfamide were different in the three-drug regimens under testing in accordance to previous phase II experience. In addition, the RR observed in this trial observed with triplets was lower than that reported in phase II studies probably as consequence of less stringent eligibility criteria and radiological response assessment.

Overall, the outcome data obtained in the FAST trial population were good; in particular, a median survival of 10.3 months was observed in the overall population, which compares favourably with that of the more relevant randomised phase III trials performed with different platinum doublets, where the median OS ranged between 7.4 and 9.9 months (Kelly *et al*, 2001; Scagliotti *et al*, 2002; Schiller *et al*, 2002). Moreover, all outcome measures were similar in squamous and non-squamous histology subtypes, suggesting the lack of treatment–histology interactions with the chemotherapy regimens used in this study.

With the limitations of long study duration, this trial confirms that in advanced NSCLC patients with a PS of 0 or 1, a two-drug platinum-based combination, such as cisplatin–gemcitabine, should be preferred as first-line treatment. The results of this trial along with those of platinum *vs* non-platinum meta-analysis and of carboplatin *vs* cisplatinum meta-analysis, suggest that cisplatin should remain a fundamental ingredient of first-line chemotherapy of advanced NSCLC patients, especially when a long-term survival can be anticipated, such as in fit patients with oligometastatic disease. Non-platinum combinations, however, can remain a reasonable option in patients who have contraindications to platinum therapy or those who are unfit or have very advanced bulky and multimetastic disease.

On the contrary, our study, in keeping with the results of a meta-analysis, further supports the concept that, at the moment, there is no place for increasing the number of chemotherapy agents beyond two in first-line regimens in the treatment of advanced NSCLC. Perhaps, the use of triplets might deserve further investigation in locally advanced non-metastatic disease where the increased RR associated with this strategy, as seen in some other studies, might lead to an improvement of tumour down-staging and, ultimately, to a better local control with subsequent locoregional therapies.

Although the currently most employed drug combinations for first-line treatment of advanced NSCLC, such as cisplatinum–pemetrexed and carboplatin-paclitaxel-bevacizumab (Sandler *et al*, 2006; Scagliotti *et al*, 2008), were not included in this study, we believe that our results can contribute further to the clarification of the optimal chemotherapy management of advanced NSCLC.

## Figures and Tables

**Table 1:** Baseline patient and tumour characteristics by trial interventions

	**Platinum-based regimen**				**Number of drugs**			
	**Yes (*N*=216)**		**No (*N*=217)**		**2-Drugs (*N*=212)**		**3-Drugs (*N*=221)**	
	**No.**	**%**	**No.**	**%**	**No.**	**%**	**No.**	**%**
Median age (range), years	63 (29–79)		64 (35–77)		62.5 (29–77)		64 (41–79)	
								
*Gender*								
Male	170	79	176	81	170	80	176	80
Female	46	21	41	19	42	20	45	20
								
*ECOG* *PS*								
0	132	61	132	61	128	60	136	62
1	74	34	77	35	75	35	76	34
2	10	5	8	4	9	4	9	4
								
*Stage*								
IIIB	46	21	42	19	45	21	43	19
IV	170	79	175	81	167	79	178	81
								
*Method of diagnosis*								
Histology	145	67	144	66	137	65	152	69
Cytology	62	29	63	29	65	31	60	27
Missing value	9	4	10	5	10	4	9	4
								
*Histology*								
Adenocarcinoma	90	42	92	42	90	42	92	42
Squamous	59	27	60	28	55	26	64	29
Large cell	6	3	2	1	4	2	4	2
NOS	61	28	63	29	63	30	61	27

Abbreviations: ECOG=Eastern Cooperative Oncology Group; NOS=not otherwise specified; PS=performance status.

**Table 2:** NCIC/CTC version 2.0 grade 3 and 4 toxicities exceeding 5% of patients in the treated patients

	**Platinum-based regimen**					**Number of drugs**				
	**Yes (*N*=207)**		**No (*N*=210)**			**2-Drugs (*N*=203)**		**3-Drugs (*N*=214)**		
	**No.**	**%**	**No.**	**%**	***P-*value**	**No.**	**%**	**No.**	**%**	***P-*value**
Anaemia	30	14	10	5	0.001	16	8	24	11	0.254
Leucopenia	69	33	49	23	0.025	44	22	74	35	0.003
Neutropenia	92	44	77	37	0.095	74	36	95	44	0.091
Thrombocytopenia	67	32	8	4	<0.001	33	16	42	20	0.339
Nausea and vomiting	24	12	8	4	0.004	16	8	16	7	0.890
Fatigue	27	13	16	8	0.074	19	9	24	11	0.507

**Table 3:** Response and survival outcomes by trial interventions

	**Platinum-based regimen**				**Number of drugs**			
	**Yes (*N*=216)**		**No (*N*=217)**		**2-Drugs (*N*=212)**		**3-Drugs (*N*=221)**	
	**No.**	**%**	**No.**	**%**	**No.**	**%**	**No.**	**%**
*Best overall response* a								
CR	4	2	4	2	4	2	4	2
PR	62	29	48	22	57	27	53	26
SD	77	36	71	33	69	33	79	35
PD	29	14	39	18	34	16	34	16
NE	41	19	52	24	47	22	46	22
								
Adjusted percentage of CR+PR (95% CI)	31% (25–37%)		24% (19–30%)		29% (23–35%)		26% (21–33%)	
Adjusted OR (95% CI), *P*-value	0.72 (0.47–1.10), *P*=0.124				0.86 (0.56–1.32), *P*=0.487			
								
*PFS*								
Number of events	201		213		201		213	
1-Year and 2-year adjusted probability	19.7% and 7.6%		12.9% and 3.9%		15.8% and 5.3%		16.5% and 5.7%	
Adjusted median (95% CI), months	6.4 (5.3–7.1)		4.9 (4.4–5.8)		5.6 (4.7–6.6)		5.7 (4.8–6.7)	
Adjusted HR (95% CI), *P*-value	1.26 (1.04–1.53), *P*=0.020				0.98 (0.81–1.19), *P*=0.820			
								
*OS*								
Number of events	190		209		195		204	
1-Year and 2-year adjusted probability	47.9% and 20.7%		40.6% and 14.5%		44.8% and 17.9%		43.8% and 17.0%	
Adjusted median (95% CI), months	11.3 (9.8–12.7)		9.7 (8.7–10.8)		10.4 (9.4–12.2)		10.3 (9.2–11.8)	
Adjusted HR (95% CI), *P*-value	1.23 (1.01–1.49), *P*=0.044				1.03 (0.85–1.25), *P*=0.781			
								

Abbreviations: CI=confidence interval; CR=complete response; HR=hazard ratio; NE=not evaluated; OR=odds ratio; OS=overall survival; PD=progressive disease; PFS=progression-free survival; PR=partial response; SD=stable disease.

a Six patients without measurable disease at randomisation were excluded from the analysis of best overall response.
