# Supplementary material for: Triplets versus doublets, with or without cisplatin, in the first-line treatment of stage IIIB–IV non-small cell lung cancer (NSCLC) patients: a multicenter randomised factorial trial (FAST)
Source: Br J Cancer. 2012 Jan 12;106(4):658–65. doi: 10.1038/bjc.2011.606 (PMC3322957; doi:10.1038/bjc.2011.606)
Supplement: Supplementary Figure S1 [file bjc2011606x1.ppt]

## Slide 1
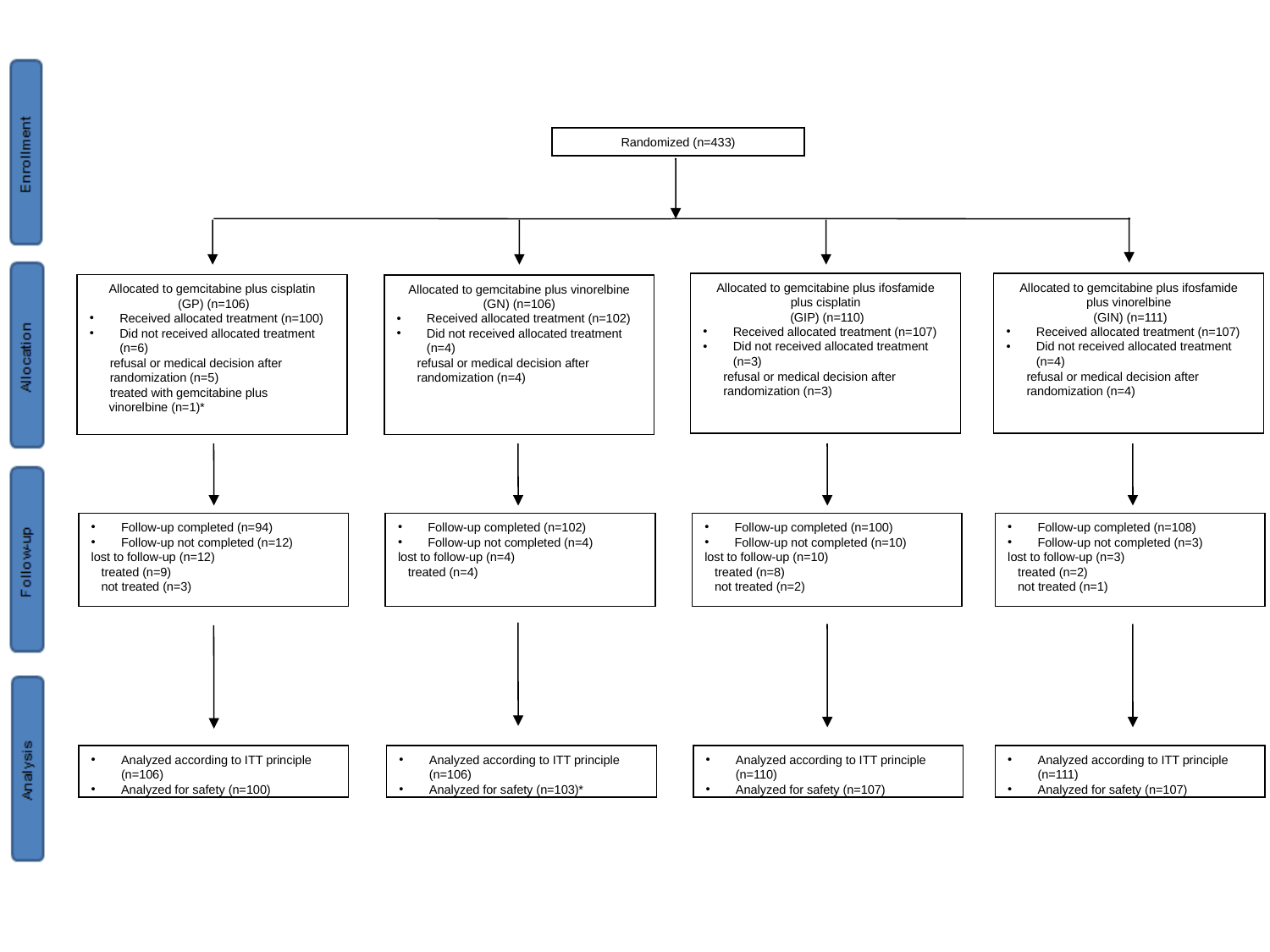

Randomized (n=433)
Allocated to gemcitabine plus ifosfamide
plus cisplatin
 (GIP) (n=110)
Received allocated treatment (n=107)
Did not received allocated treatment (n=3)
 refusal or medical decision after
 randomization (n=3)
Allocated to gemcitabine plus ifosfamide
plus vinorelbine
 (GIN) (n=111)
Received allocated treatment (n=107)
Did not received allocated treatment (n=4)
 refusal or medical decision after
 randomization (n=4)
Allocated to gemcitabine plus cisplatin
 (GP) (n=106)
Received allocated treatment (n=100)
Did not received allocated treatment (n=6)
 refusal or medical decision after
 randomization (n=5)
 treated with gemcitabine plus 	 vinorelbine (n=1)*
Allocated to gemcitabine plus vinorelbine (GN) (n=106)
Received allocated treatment (n=102)
Did not received allocated treatment (n=4)
 refusal or medical decision after
 randomization (n=4)
Follow-up completed (n=94)
Follow-up not completed (n=12)
lost to follow-up (n=12)
 treated (n=9)
 not treated (n=3)
Follow-up completed (n=102)
Follow-up not completed (n=4)
lost to follow-up (n=4)
 treated (n=4)
Follow-up completed (n=100)
Follow-up not completed (n=10)
lost to follow-up (n=10)
 treated (n=8)
 not treated (n=2)
Follow-up completed (n=108)
Follow-up not completed (n=3)
lost to follow-up (n=3)
 treated (n=2)
 not treated (n=1)
Analyzed according to ITT principle (n=106)
Analyzed for safety (n=100)
Analyzed according to ITT principle (n=106)
Analyzed for safety (n=103)*
Analyzed according to ITT principle (n=110)
Analyzed for safety (n=107)
Analyzed according to ITT principle (n=111)
Analyzed for safety (n=107)
