# Supplementary material for: Triplets versus doublets, with or without cisplatin, in the first-line treatment of stage IIIB–IV non-small cell lung cancer (NSCLC) patients: a multicenter randomised factorial trial (FAST)
Source: Br J Cancer. 2012 Jan 12;106(4):658–65. doi: 10.1038/bjc.2011.606 (PMC3322957; doi:10.1038/bjc.2011.606)
Supplement: Supplementary Figure S2 [file bjc2011606x2.ppt]

## Slide 1
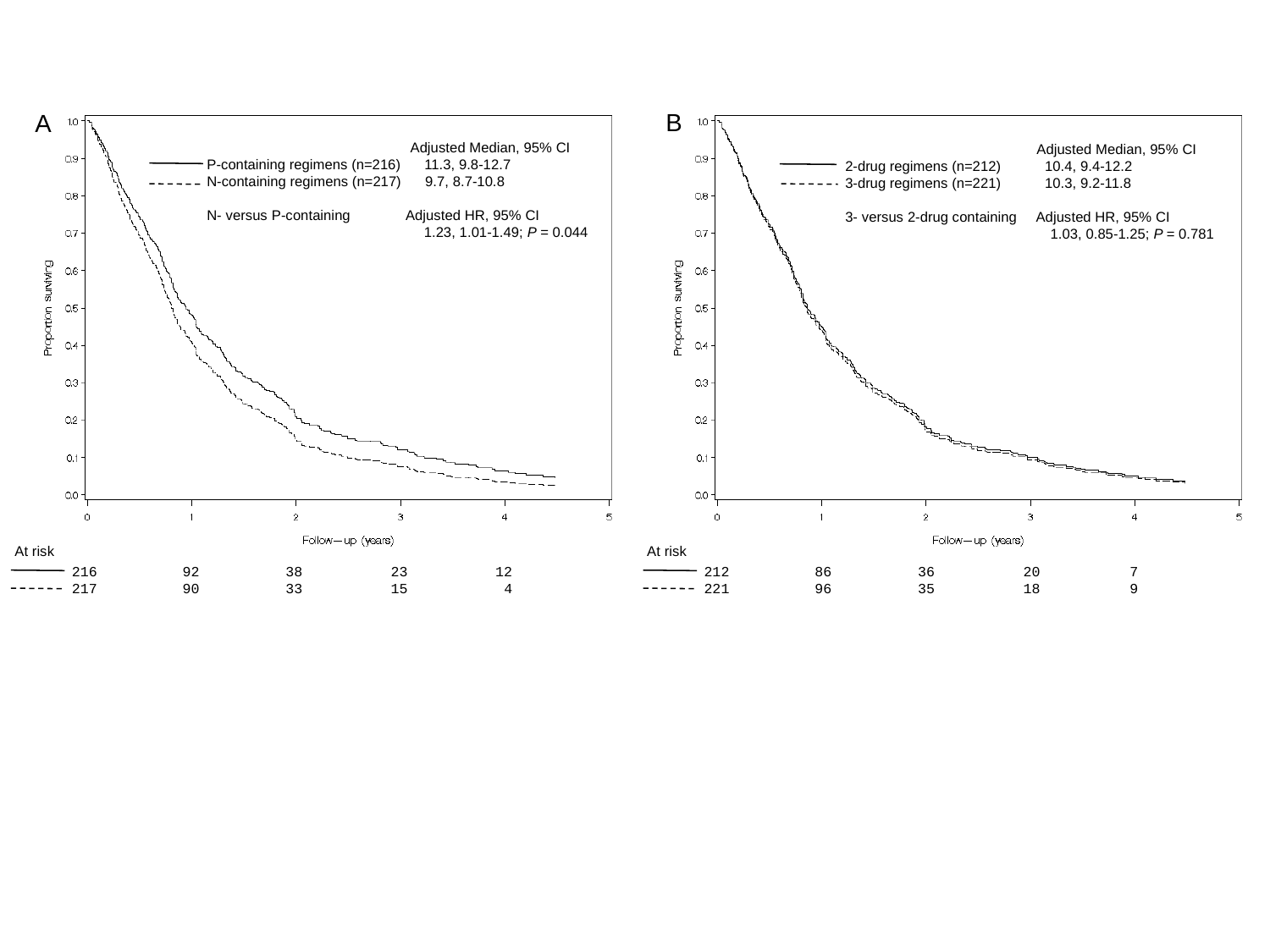

B
A
 	 Adjusted Median, 95% CI
P-containing regimens (n=216) 11.3, 9.8-12.7
N-containing regimens (n=217) 9.7, 8.7-10.8
N- versus P-containing Adjusted HR, 95% CI
 1.23, 1.01-1.49; P = 0.044
 	 Adjusted Median, 95% CI
2-drug regimens (n=212) 10.4, 9.4-12.2
3-drug regimens (n=221) 10.3, 9.2-11.8
3- versus 2-drug containing Adjusted HR, 95% CI
 1.03, 0.85-1.25; P = 0.781
At risk
At risk
216
217
92
90
38
33
23
15
12
4
212
221
86
96
36
35
20
18
7
9

## Slide 2
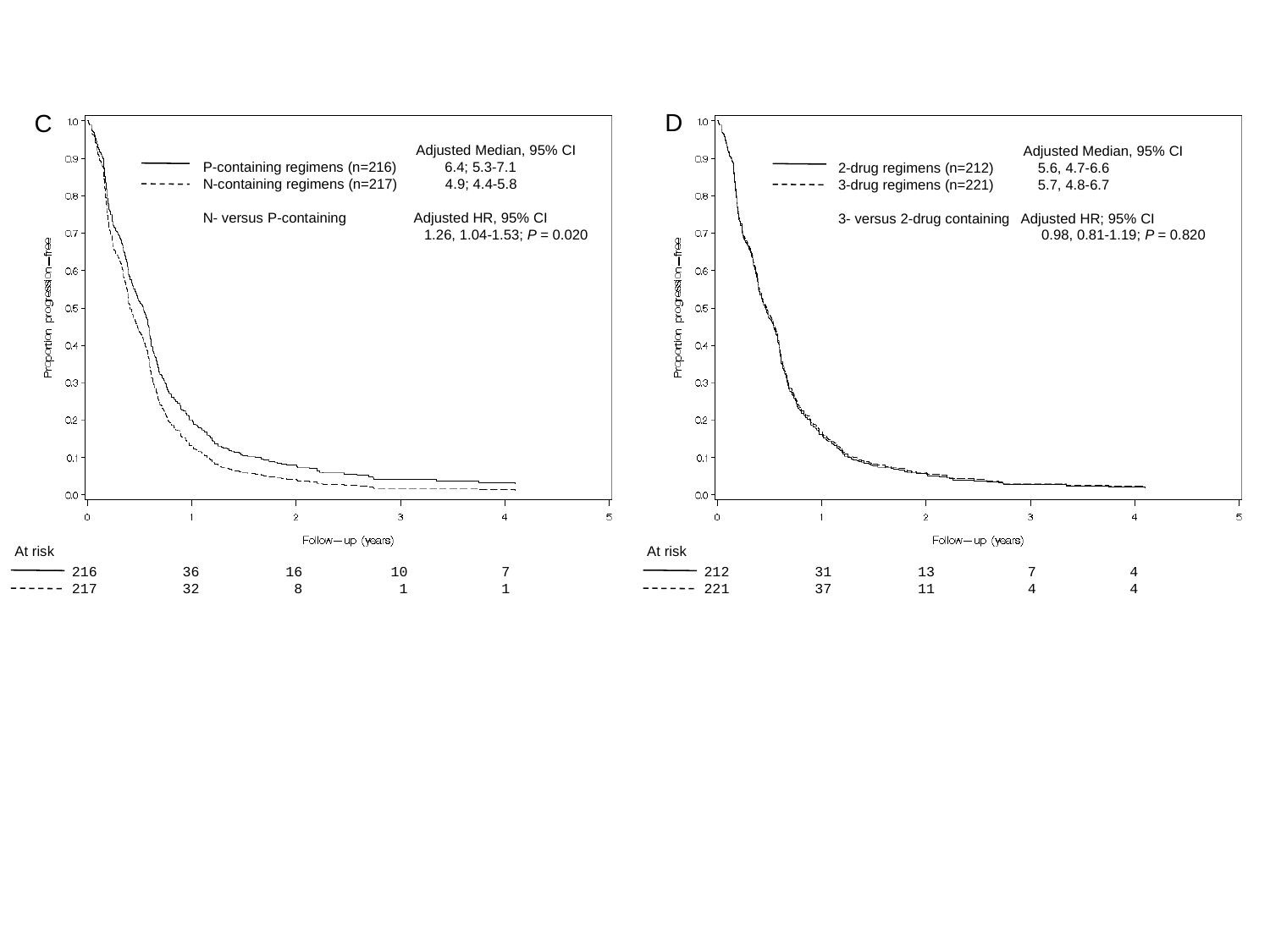

D
C
 Adjusted Median, 95% CI
P-containing regimens (n=216) 6.4; 5.3-7.1
N-containing regimens (n=217) 4.9; 4.4-5.8
N- versus P-containing Adjusted HR, 95% CI
 1.26, 1.04-1.53; P = 0.020
 Adjusted Median, 95% CI
2-drug regimens (n=212) 5.6, 4.7-6.6
3-drug regimens (n=221) 5.7, 4.8-6.7
3- versus 2-drug containing Adjusted HR; 95% CI
	 0.98, 0.81-1.19; P = 0.820
At risk
At risk
216
217
36
32
16
8
10
1
7
1
212
221
31
37
13
11
7
4
4
4
