# Supplementary material for: Triplets versus doublets, with or without cisplatin, in the first-line treatment of stage IIIB–IV non-small cell lung cancer (NSCLC) patients: a multicenter randomised factorial trial (FAST)
Source: Br J Cancer. 2012 Jan 12;106(4):658–65. doi: 10.1038/bjc.2011.606 (PMC3322957; doi:10.1038/bjc.2011.606)
Supplement: Supplementary Tables [file bjc2011606x3.doc]

**Table S1**. Baseline patient and tumor characteristics by allocated treatment arm.

|  | **Allocated treatment arm** | | | | | | | |
| --- | --- | --- | --- | --- | --- | --- | --- | --- |
|  | **GP**  **(N=106)** | | **GN**  **(N=106)** | | **GIP**  **(N=110)** | | **GIN**  **(N=111)** | |
|  | **No.** | **%** | **No.** | **%** | **No.** | **%** | **No.** | **%** |
|  |  |  |  |  |  |  |  |  |
| Median age [range], years | 61 [29-77] | | 63.5 [35-77] | | 64 [41-79] | | 64 [42-77] | |
|  |  |  |  |  |  |  |  |  |
| Gender |  |  |  |  |  |  |  |  |
| Male | 87 | 82 | 83 | 78 | 83 | 75 | 93 | 84 |
| Female | 19 | 18 | 23 | 22 | 27 | 25 | 18 | 16 |
|  |  |  |  |  |  |  |  |  |
| ECOG PS |  |  |  |  |  |  |  |  |
| 0 | 60 | 56 | 68 | 64 | 72 | 65 | 64 | 58 |
| 1 | 42 | 40 | 33 | 31 | 32 | 29 | 44 | 39 |
| 2 | 4 | 4 | 5 | 5 | 6 | 6 | 3 | 3 |
|  |  |  |  |  |  |  |  |  |
| Stage |  |  |  |  |  |  |  |  |
| IIIB | 22 | 21 | 23 | 22 | 24 | 22 | 19 | 17 |
| IV | 84 | 79 | 83 | 78 | 86 | 78 | 92 | 83 |
|  |  |  |  |  |  |  |  |  |
| Method of diagnosis |  |  |  |  |  |  |  |  |
| Histology | 67 | 63 | 70 | 66 | 78 | 71 | 74 | 67 |
| Cytology | 34 | 32 | 31 | 29 | 28 | 25 | 32 | 29 |
| Missing value | 5 | 5 | 5 | 5 | 4 | 4 | 5 | 4 |
|  |  |  |  |  |  |  |  |  |
| Histology |  |  |  |  |  |  |  |  |
| Adenocarcinoma | 45 | 42 | 45 | 43 | 45 | 41 | 47 | 42 |
| Squamous | 26 | 24 | 29 | 27 | 33 | 30 | 31 | 28 |
| Large cell | 3 | 3 | 1 | 1 | 3 | 3 | 1 | 1 |
| NOS | 32 | 31 | 31 | 29 | 29 | 26 | 32 | 29 |
|  |  |  |  |  |  |  |  |  |

Abbreviations: PS, performance status; NOS, not otherwise specified; GP, gemcitabine-cisplatin; GN, gemcitabine-vinorelbine; GIP, gemcitabine-ifosfamide-cisplatin; GIN gemcitabine-ifosfamide-vinorelbine.

**Table S2**. NCIC/CTC version 2.0 grade 3 and 4 toxicities exceeding 5% of patients by allocated treatment arm.

|  | **Allocated treatment arm** | | | | | | | |
| --- | --- | --- | --- | --- | --- | --- | --- | --- |
|  | **GP**  **(N=100)** | | **GN**  **(N=103)** | | **GIP**  **(N=107)** | | **GIN**  **(N=107)** | |
|  | **No.** | **%** | **No.** | **%** | **No.** | **%** | **No.** | **%** |
|  |  |  |  |  |  |  |  |  |
| Anemia | 13 | 13 | 3 | 3 | 17 | 16 | 7 | 7 |
| Leucopenia | 27 | 27 | 17 | 17 | 42 | 39 | 32 | 30 |
| Neutropenia | 41 | 41 | 33 | 32 | 51 | 48 | 44 | 41 |
| Thrombocytopenia | 31 | 31 | 2 | 2 | 36 | 34 | 6 | 6 |
| Nausea and vomiting | 11 | 11 | 5 | 5 | 13 | 12 | 3 | 3 |
| Fatigue | 12 | 12 | 7 | 7 | 15 | 14 | 9 | 8 |
|  |  |  |  |  |  |  |  |  |

GP, gemcitabine-cisplatin; GN, gemcitabine-vinorelbine; GIP, gemcitabine-ifosfamide-cisplatin; GIN gemcitabine-ifosfamide-vinorelbine.

**Table S3**. Response and survival outcomes by allocated treatment arm.

|  | **Allocated treatment arm** | | | | | | | |
| --- | --- | --- | --- | --- | --- | --- | --- | --- |
|  | **GP**  **(N=106)** | | **GN**  **(N=106)** | | **GIP**  **(N=110)** | | **GIN**  **(N=111)** | |
|  | **No.** | **%** | **No.** | **%** | **No.** | **%** | **No.** | **%** |
|  |  |  |  |  |  |  |  |  |
| Best overall response(*) |  |  |  |  |  |  |  |  |
| CR | 2 | 2 | 2 | 2 | 2 | 2 | 2 | 2 |
| PR | 33 | 31 | 24 | 23 | 29 | 27 | 24 | 22 |
| SD | 33 | 31 | 36 | 34 | 44 | 41 | 35 | 32 |
| PD | 19 | 18 | 15 | 14 | 10 | 10 | 24 | 22 |
| NE | 19 | 18 | 28 | 27 | 22 | 20 | 24 | 22 |
|  |  |  |  |  |  |  |  |  |
| Percentage of CR+PR (95% CI) | 33% (24%-43%) | | 25% (17%-34%) | | 29% (21%-39%) | | 24% (16%-33%) | |
|  |  |  |  |  |  |  |  |  |
| PFS |  |  |  |  |  |  |  |  |
| Number of events | 98 | | 103 | | 103 | | 110 | |
| 1-yr and 2-yr probability | 13.2% and 8.1% | | 17.4% and 4.8% | | 21.5% and 7.5% | | 12.7% and 2.7% | |
| Median (95% CI), months | 5.5 (4.6-6.6) | | 4.6 (3.9-6.8) | | 7.2 (6.3-7.7) | | 4.7 (3.1-5.6) | |
|  |  |  |  |  |  |  |  |  |
| OS |  |  |  |  |  |  |  |  |
| Number of events | 94 | | 101 | | 96 | | 108 | |
| 1-yr and 2-yr probability | 40.9% and 18.4% | | 45.3% and 17.7% | | 49.2% and 19.9% | | 40.8% and 13.9% | |
| Median (95% CI), months | 9.6 (8.2-12.2) | | 11.3 (8.8-12.8) | | 11.8 (9.1-15.0) | | 10.0 (6.7-12.0) | |
|  |  |  |  |  |  |  |  |  |

(*) Six patients without measurable disease at randomization were excluded from the analysis of best overall response.

Abbreviations: CR, complete response; PR, partial response; SD, stable disease; PD, progressive disease; NE, not evaluated; PFS, progression‑free survival; CI, confidence interval; OS, overall survival; GP, gemcitabine-cisplatin; GN, gemcitabine-vinorelbine; GIP, gemcitabine-ifosfamide-cisplatin; GIN gemcitabine-ifosfamide-vinorelbine.
